# GlucoseGo: a simple tool to predict hypoglycaemia during exercise in type 1 diabetes

**DOI:** 10.1007/s00125-026-06692-8

**Published:** 2026-02-25

**Authors:** Catherine L. Russon, Michael J. Allen, Emma Cockcroft, John S. Pemberton, Anne-Marie Frohock, Neil Vaughan, Richard M. Pulsford, Robert C. Andrews

**Affiliations:** 1 University of Exeter Medical School, University of Exeter, Exeter, UK; 2 Department of Endocrinology and Diabetes, Birmingham Women’s and Children’s Foundation Trust, Birmingham, UK; 3 Oxfordshire Children’s Diabetes Service, Oxford University Hospitals NHS Foundation Trust, Oxford, UK

**Keywords:** Continuous glucose monitoring, Decision support, Exercise, Hypoglycaemia, Machine learning, Risk prediction, Self-management, Type 1 diabetes

## Abstract

**Aims/hypothesis:**

This study aimed to develop an accessible tool, derived using machine learning, to predict hypoglycaemia risk at the start of exercise and to provide clear, rapid risk assessment to support safer participation in exercise.

**Methods:**

Data from four diverse studies were combined, encompassing 16,430 exercise sessions from 834 participants aged 12–80 years using various insulin delivery methods. The XGBoost algorithm was used to develop two models: a comprehensive model and a simplified model for predicting hypoglycaemia during exercise.

**Results:**

The comprehensive model (406 variables) achieved a mean ROC AUC of 0.89. The simplified model, using only starting glucose, exercise duration and glucose trend arrows, achieved a comparable ROC AUC of 0.87. The simplified model performed consistently across exercise types and insulin delivery methods. In collaboration with individuals with type 1 diabetes, this model was translated into GlucoseGo, a user-friendly traffic-light heatmap displaying hypoglycaemia risk based on the three variables.

**Conclusions/interpretation:**

The GlucoseGo heatmap provides a practical, accessible tool for predicting hypoglycaemia risk immediately before exercise. It may empower individuals with type 1 diabetes to exercise more safely, reduce hypoglycaemic episodes, and increase engagement in physical activity.

**Graphical Abstract:**

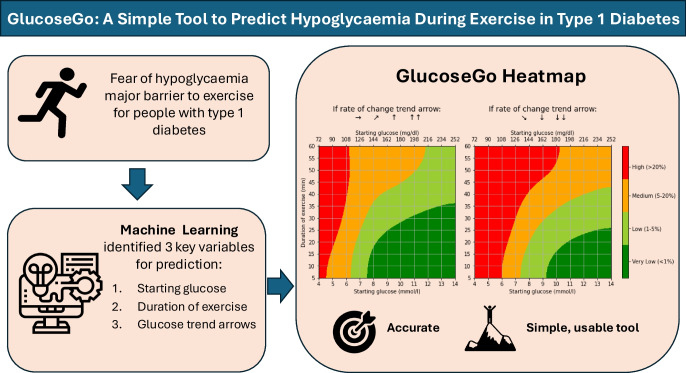

**Supplementary Information:**

The online version contains peer-reviewed but unedited supplementary material available at 10.1007/s00125-026-06692-8.


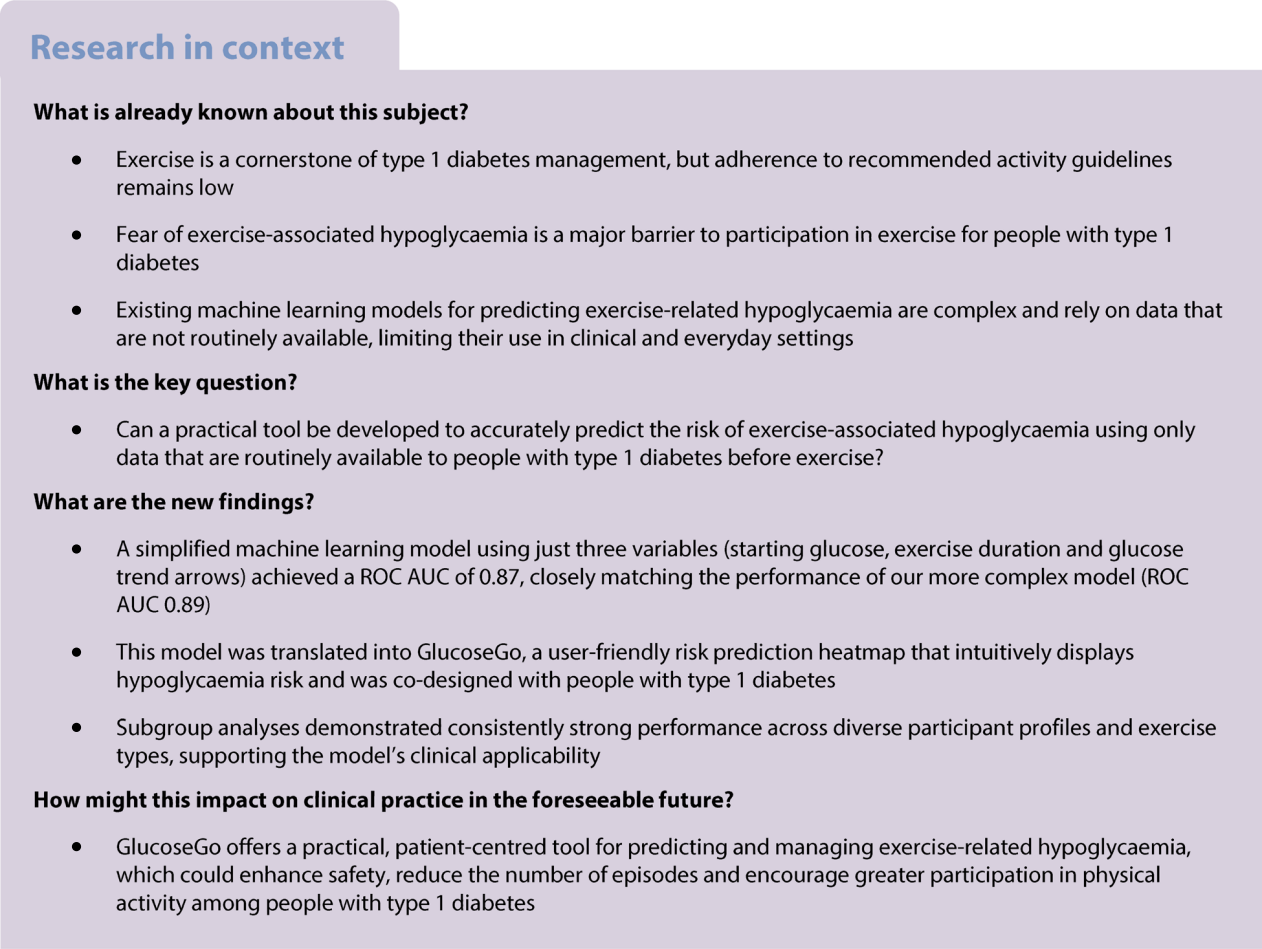


## Introduction

Exercise is crucial in the management of type 1 diabetes, providing significant benefits, including improved glycaemic management, cardiovascular health and psychological wellbeing, and reduced long-term complications [1]. Current guidelines recommend at least 150 min of moderate-intensity activity per week for adults with type 1 diabetes [2]. However, adherence remains low, with only around half of adults achieving this target [3]. Fear of exercise-associated hypoglycaemia is consistently identified as the most important barrier to participation [4]. Hypoglycaemia not only poses immediate health risks but also has a psychological impact that can discourage regular exercise, impacting overall wellbeing and compounding the long-term challenges of managing diabetes effectively [5].

In recent years, machine learning (ML) has emerged as a promising tool for predicting hypoglycaemia and supporting diabetes self-management [6]. Applying ML to exercise has particular potential: providing people with type 1 diabetes with personalised risk information before activity could allow them to take appropriate precautions or to exercise with reassurance if the risk is low, thereby improving both safety and confidence. Research to date shows promise, with three existing studies effectively predicting hypoglycaemia during exercise [7–9]. However, these models are often complex and rely on specialised data inputs that individuals with type 1 diabetes do not readily have, limiting their practical applicability outside research settings and resulting in minimal adoption by patients and clinicians.

To address these limitations, we aimed to develop a predictive model that combined accuracy with simplicity. Our objective was to create and validate a streamlined approach that avoided the complexity of previous ML-based tools, with the intention of producing a practical, user-friendly resource to help people with type 1 diabetes quickly assess their risk of hypoglycaemia before exercise and to support safer, more confident participation in physical activity.

## Methods

**Fig. 1:**
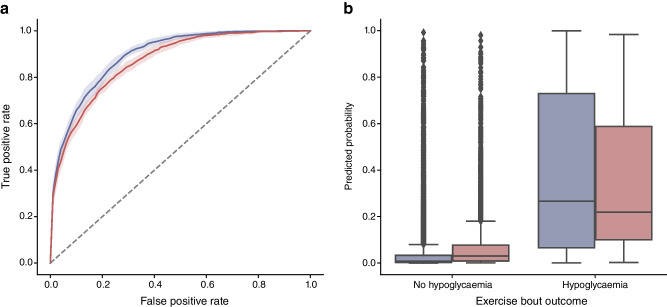
Accuracy measures for predicting hypoglycaemia during exercise. (**a**) ROC curves for the complex (blue) and simplified (red) XGBoost models, with shaded areas indicating 95% CI. The dashed line indicates the mean decision threshold used for binary classification. (**b**) Boxplots showing the distribution of predicted hypoglycaemia probabilities for individual exercise bouts for both models, stratified by observed outcome (hypoglycaemia vs no hypoglycaemia). The horizontal line represents the median, boxes indicate the IQR, whiskers extend to 1.5 × IQR, and diamond symbols indicate outliers

An overview of the workflow is provided in electronic supplementary material (ESM) Fig. 1, together with more detailed methods regarding building of the ML model. All data analysis and processing were conducted using pandas (version 1.3) [10] and NumPy (version 1.21) [11] in Python (version 3.9) and (R version 4.5). Figures were created using Matplotlib (version 3.5) [12]. All code for this project is openly available in a GitHub repository (https://github.com/cafoala/glucoseGo). Development and validation of ML models were conducted in accordance with the TRIPOD framework [13].

### Data source and participants

This study is a retrospective cohort analysis using four datasets. Two of these come from the EXercise in Type One Diabetes (EXTOD) group (https://www.extod.org/) and two from the Jaeb Center for Health Research (JCHR) (https://www.jaeb.org/).

The JCHR datasets are from two large observational exercise studies, one in adults [14] and one in young people aged 12–17 years [15]. In the former, a total of 561 adult participants in the Type 1 Diabetes EXercise Initiative (T1DEXI) study provided 4 weeks of free-living glucose, insulin and exercise data. In the latter, a total of 251 young people participating in the Type 1 Diabetes EXercise Initiative Pediatric (T1DEXIP) study provided 10 days of free-living glucose, insulin and exercise data. In both cohorts, participants wore an unblinded Dexcom G6 continuous glucose monitor (CGM) (Dexcom) and recorded exercise on a fitness tracker (Verily Study Watch [Verily Life Sciences] for adults and Garmin Vivosmart [Garmin] for young people). Full details of the exercise protocols and data collection procedures are provided elsewhere [14, 15].

The EXTOD-101 (EXT-101) study [16] followed 69 adults with type 1 diabetes who were training for a long-distance running race. Glucose was recorded using a FreeStyle Libre CGM, (Abbott Diabetes Care) and exercise bouts were recorded in an exercise diary for 6 weeks before the race to 3 weeks after the race. The EXTOD-Education (EXT-EDU) study [17] assessed whether a type 1 diabetes educational programme improved glucose management around exercise, with 2 weeks of data for 98 participants: 1 week at baseline and 1 week at 6 months post-intervention. Glucose was recorded using a blinded Dexcom G6 CGM, and exercise bouts were recorded in an exercise diary. No insulin data were available for either EXTOD study.

All studies received institutional ethics approval and were conducted in accordance with the Declaration of Helsinki (2024 revision).

### Inclusion criteria

Exercise bouts were excluded if we could not determine the precise date/time that they occurred (*n*=71), they were duplicated or overlapping (*n*=831), they were outside the range of 10–120 min in duration (*n*=2353), there was incomplete corresponding CGM data (<60% data sufficiency during bout and <40% data sufficiency for the hour prior) (*n*=2744), or the reported ‘exercise’ bouts were actually activities of daily living or occupational activities rather than volitional exercise as defined by Caspersen et al [18] (*n*=1167) (ESM Fig. 1). The resulting dataset consisted of 16,430 exercise bouts.

### Predictor and target variables

Hypoglycaemia was the target variable (i.e. the outcome that the ML models were trained to predict) and was determined using CGM data. It was defined as any readings below the threshold of level 1 hypoglycaemia (3.9 mmol/l [70 mg/dl]) during the exercise bout, rather than requiring a sustained hypoglycaemic episode of ≥15 min. This threshold was chosen because, in real-world settings, individuals typically perceive and act on a single low reading of 3.9 mmol/l, often consuming carbohydrate or pausing activity before hypoglycaemia persists for long enough to meet episode-based definitions.

We assessed the following predictor variables: age, biological sex (clinician-recorded or genotype-verified), race (self-reported), BMI (kg/m^2^), years since diagnosis, method of insulin administration (multiple daily injections [MDI], insulin pump or closed-loop system), HbA_1c_ (mmol/mol), the exercise start time (morning, afternoon or evening), exercise duration (min), the self-reported type of exercise (converted to predominantly aerobic, anaerobic or mixed by a study author with expertise in sport and exercise science) and the self-reported intensity.

Exercise intensity was assessed differently across the four studies. In the T1DEXI and T1DEXIP cohorts, participants selected one of three options (light, moderate or vigorous) using the study app or activity tracker. In contrast, the EXT-101 and EXT-EDU studies recorded intensity using the Borg rating of perceived exertion scale (6–20) [19]. Borg values were cleaned and standardised, and then converted into the same three intensity categories using commonly applied rate of perceived effort (RPE) thresholds, with scores <12 classified as light, scores of 12–14 classified as moderate and scores >14 classified as vigorous.

For insulin administration, we extracted two variables: insulin units on board per kg (IOB/kg) and time since last insulin bolus dose (rounded to the nearest hour). Doses for pump and automated insulin delivery users were obtained from device downloads, whereas MDI users manually entered bolus doses into the study application. The IOB/kg was calculated using a linear degradation algorithm with the duration of insulin action set to 4 h. IOB was calculated at 1 min intervals to provide a continuous time-varying estimate of active insulin. The same procedure was applied across all therapy modalities (MDI, pump and closed-loop), ensuring comparability of IOB estimates between participants. Temporary basal reductions or suspensions were not modelled separately.

Carbohydrate consumption immediately before or during exercise was not included as a predictor because, as shown in our previous work [20], it represents a behavioural response to perceived hypoglycaemia risk rather than an independent exposure, and therefore lies on the treatment pathway from risk to outcome.

The starting glucose was determined from CGM data (last reading before the start of exercise), and the glucose rate of change was computed using the difference between this reading and the glucose reading 15 min before. The cut-offs for glucose rate of change were falling (< −0.05 mmol l^−1^ min^−1^ [< −0.9 mg dl^−1^ min^−1^]), stable (−0.05 to 0.05 mmol l^−1^ min^−1^ [−0.9 to 0.9 mg dl^−1^ min^−1^]) and rising (>0.05 mmol l^−1^ min^−1^ [>0.9 mg dl^−1^ min^−1^]) [21]. Glucose trend arrows were calculated from rate of change and standardised across CGM systems using the equivalence framework described by Miller et al [21]. For the 1 h CGM period prior to exercise, we used Diametrics *(*version 0.4) [22] to extract standard metrics of glycaemic management (e.g. time in normal range, mean glucose and coefficient of variation) [23] and tsfresh (version 0.19) [24] to extract advanced statistical features from the CGM data.

To fill missing data, we used multivariate imputation using a *k*-nearest neighbour (*k-*nn) algorithm (*k*=5).

Ultimately, our dataset comprised 406 distinct variables (ESM Table 1).

### Model implementation and evaluation

We used XGBoost (eXtreme Gradient Boosting algorithm) as the ML algorithm for this analysis. XGBoost is a decision tree-based ensemble learning method that is known for its accuracy, generalisability and ability to capture nonlinearity and interactions between features [25].

The T1DEXI dataset (*N*=12,450) was used to build the models using stratified *k*-fold cross-validation (*k*=10) (ESM Fig. 2 and ESM Methods). To account for the repeated-measures structure in the dataset (whereby multiple exercise events were recorded for each participant), participants, rather than individual exercise events, were grouped within each fold, preserving within-participant correlations and preventing data leakage between folds. The models were then optimised using Bayesian hyperparameter tuning [26, 27]. The tuned hyperparameters for the model are available in ESM Table 2.

Performance of the models was primarily assessed using the area under the receiver operating characteristics curve (ROC AUC) and calibration curves. In addition, we used thresholding to calculate sensitivity and specificity.

### Creating a simplified model

We identified 13 variables that are readily available to people with type 1 diabetes as they are about to begin exercise: the type of exercise (aerobic, anaerobic or mixed), the duration of exercise, the intensity, the time of day (morning: 05:00–12:00 hours; afternoon: 12:00–17:00 hours; evening: 17:00–05:00 hours), starting glucose, glucose trend arrows, time since last insulin bolus (<1.5 h ago, 1.5–3.5 h ago or >3.5 h ago), age, method of insulin administration, years since diagnosis, sex, BMI and HbA_1c_. Using forward feature selection with XGBoost [28], we iteratively assessed each feature’s contribution to model performance based on the ROC AUC. To prioritise simplicity and usability as a visual tool, only the variables that our patient and clinical group considered to provide meaningful performance improvements without adding unnecessary complexity were selected. The model was then trained with only these selected variables, following the methods described above (Methods/Model implementation and evaluation). The tuned hyperparameters for the model are available in ESM Table 3.

### Assessing transferability to other populations

To test the transferability of the simplified model across populations, we validated it on the three other cohorts: T1DEXIP (*N*=3132), EXT-101 (*N*=422) and EXT-EDU (*N*=426). For each cohort, the *k*-fold (*k*=10) simplified model was applied, and the mean prediction and 95% CI across folds were calculated to obtain a robust performance measure for each dataset.

The performance of the model on these separate cohorts was again evaluated using ROC AUC curves and sensitivity and specificity.

### Model transparency

We used SHapley Additive exPlanations (SHAP) values to enhance the interpretability of XGBoost's ‘black box’ decisions. This method assigns importance values to variables for individual predictions, providing a clearer understanding of their impact on the model's outcomes [29].

We also conducted a subgroup analysis, calculating observed and predicted hypoglycaemia rates and ROC AUC, and visualised the results [30]. The subgroups assessed were starting glucose, duration of exercise, starting rate of change, IOB/kg, age, sex, insulin administration, insulin modality, type of exercise, intensity of exercise, years since diagnosis, HbA_1c_ and coefficient of variation. Subgroup analysis across racial groups was not possible due to limited numbers in some of the subgroups.

## Results

### Participant characteristics

The characteristics of the participants are shown in Table 1. In total, there were 834 participants, of which 61% were female. The median age was 27 years with a median duration of diabetes of 12 years. Closed-loop systems were the most common insulin delivery modality. Overall glycaemic management was close to recommended targets, with a median HbA_1c_ of 50.8 mmol/mol (6.8%) (target 53 mmol/mol [7%]) and median time in range (3.9–10 mmol/l) of 72.2% (target 70%) [31].

**Table 1:** Characteristics of participants included in the study

Variable	All	T1DEXI	T1DEXIP	EXT-101	EXT-EDU
Demographic characteristics					
Number of participants	834	496	246	34	58
Age (years)	27.0 (16.0–42.0)	33.0 (25.0–46.0)	14.0 (12.0–15.0)	45.2 (37.7–51.1)	47.5 (36.3–56.8)
BMI (kg/m^2^)	23.7 (21.3–26.3)	24.5 (22.7–27.1)	20.7 (18.6–23.6)	23.5 (22.2–25.2)	25.0 (22.8–29.3)
HbA_1c_ (mmol/mol)	50.8 (44.3–57.0)	48.6 (43.2–54.1)	53.0 (44.3–59.6)	57.0 (51.3–61.0)	60.0 (54.0–65.8)
HbA_1c_ (%)	6.8 (6.2–7.4)	6.6 (6.1–7.1)	7.0 (6.2–7.6)	7.4 (6.9–7.7)	7.6 (7.1–8.2)
CGM device		Unblinded Dexcom G6	Unblinded Dexcom G6	Unblinded FreeStyle Libre	Blinded Dexcom G6
Insulin modality					
MDI	196	87	36	15	58
Insulin pump	279	187	73	19	0
Closed-loop	359	222	137	0	0
Years since diagnosis	12.0 (9.0–21.0)	16.0 (11.0–24.0)	10.0 (7.0–12.0)	19.1 (10.2–32.9)	14.0 (4.5–23.3)
Sex					
Female	509	362	104	20	23
Male	325	134	142	14	35
Race					
White	764	453	219	34	58
Asian	15	10	5 (2)	0	0
Black/African American	12	10	2	0	0
American Indian/Alaskan Native	3	2	1	0	0
Multiple	21	8	13	0	0
Unknown	19	13	6	0	0
Glycaemic management					
Mean glucose (mmol/l)	8.3 (7.5–9.6)	7.9 (7.2–8.8)	8.8 (7.8–10.0)	8.8 (7.9–9.9)	10.3 (9.0–11.1)
Glucose standard deviation (mmol/l)	2.9 (2.3–3.6)	2.6 (2.2–3.1)	3.1 (2.5–3.8)	3.6 (3.2–4.2)	4.0 (3.5–4.7)
Coefficient of variation (%)	34.2 (30.0–38.4)	33.0 (29.1–36.9)	34.6 (30.8–39.2)	40.3 (35.9–46.2)	37.8 (34.8–42.7)
TIR (normal range) (%)	72.2 (57.2–82.1)	76.9 (65.9–84.9)	68.2 (55.8–79.2)	60.0 (48.6–66.4)	47.3 (40.2–61.7)
TIR (tight range) (%)	46.2 (33.2–59.2)	51.0 (39.9–62.0)	44.5 (30.3–55.4)	37.2 (29.2–44.6)	27.6 (22.0–37.1)
TIR level 1 hypoglycaemia (%)	2.4 (1.0–4.4)	2.4 (1.1–4.5)	2.1 (0.7–3.7)	4.4 (1.8–9.7)	2.7 (1.1–5.4)
TIR level 2 hypoglycaemia (%)	0.3 (0.1–0.8)	0.3 (0.1–0.7)	0.2 (0.0–0.6)	1.1 (0.4–4.0)	0.6 (0.1–2.1)
TIR level 1 hyperglycaemia (%)	24.3 (14.0–39.2)	19.7 (11.1–30.8)	29.1 (17.9–42.0)	32.8 (23.4–45.2)	47.8 (31.8–56.0)
TIR level 2 hyperglycaemia (%)	4.7 (1.4–12.0)	3.0 (0.9–6.8)	7.7 (2.7–14.9)	9.3 (4.9–17.9)	18.6 (8.9–27.7)
Exercise					
Method for recording exercise		Verily Study Watch	Garmin Vivosmart	Paper diaries	Paper diaries
Total number of bouts	16,430	12,450	3132	422	426
Bouts per person	17.0 (10.0–26.0)	23.0 (16.0–31.0)	11.0 (8.0–16.0)	10.5 (6.25–18.0)	5.0 (2.25–11.0)
Bouts experiencing hypoglycaemia (%)	9.4	9.0	10.7	9.5	11.7
Start glucose (mmol/l)	7.8 (6.2–10.1)	7.7 (6.1–9.7)	8.3 (6.3–11.1)	8.8 (6.7–12.1)	8.7 (6.7–11.8)
Duration (min)	30.0 (22.0–48.0)	30.0 (22.0–45.0)	35.0 (20.0–60.0)	58.0 (40.0–60.0)	55.0 (40.0–60.0)
Time of day					
Morning	5582	4457	756	179	190
Afternoon	5679	4019	1476	65	119
Evening	5169	3974	900	178	117
Intensity					
Light	7142	4882	1867	167	226
Moderate	6997	5910	785	160	142
Vigorous	2091	1658	322	84	27
Predominant form of exercise					
Aerobic	10,610	7641	2282	335	352
Anaerobic	1756	1505	166	30	55
Mixed	4064	3304	684	57	19

Data for continuous variables are medians (IQR); data for categorical variables indicate number of participants

Separate characteristics for the four study cohorts are provided, together with their aggregated characteristics ('All')

TIR, time in range; TIR (normal range): 3.9–10.0 mmol/l; TIR (tight range): 3.9–7.8 mmol/l; TIR hypoglycaemia (level 1): <3.9 mmol/l; TIR hypoglycaemia (level 2): <3.0 mmol/l; TIR hyperglycaemia (level 1): >10.0 mmol/l; TIR hyperglycaemia (level 2): >13.9 mmol/l

A total of 16,430 exercise sessions were included, with each participant logging a median of 17 sessions. The exercise sessions had a median duration of 30 min, were predominantly aerobic (65%) and most commonly of light intensity (43%). Hypoglycaemia occurred in 9.4% of all exercise sessions, a rate that was consistent across all studies. Notably, participants from the EXTOD cohorts were generally older than those in the adult JCHR cohort, used MDI more frequently, and had higher HbA_1c_ levels and a lower time in range compared with the two JCHR cohorts.

### Model development: forward variable selection and simplified XGBoost model

The results for forward variable selection are shown in Table 2. Starting glucose was identified as the strongest individual predictor of hypoglycaemia during exercise, achieving a ROC AUC of 0.780. Incorporating exercise duration improved performance by 0.048, and the addition of a ‘falling’ glucose trend arrow contributed a further increase of 0.023, yielding a combined ROC AUC of 0.851.

**Table 2:** Variable selection for the XGBoost model, showing the 13 variables identified as readily available to individuals with type 1 diabetes, ordered by importance, as determined through forward variable selection

Variables included	ROC AUC score	Change in ROC AUC score
Starting glucose (mmol/l)	0.780	0.078
Plus duration (min)	0.828	0.048
Plus rate of change Falling	0.851	0.023
Plus time since last insulin >3.5 h	0.862	0.011
Plus time since last insulin <1.5 h	0.865	0.003
Plus type of exercise Aerobic	0.867	0.002
Plus time of day Morning	0.871	0.004
Plus sex	0.873	0.002
Plus time of day Evening	0.871	−0.002
Plus rate of change Stable	0.871	0
Plus type of exercise Mixed	0.871	0
Plus insulin modality Closed-loop	0.869	−0.002
Plus insulin modality Pump	0.868	−0.001
Plus exercise intensity	0.865	−0.003
Plus age (years)	0.853	−0.012
Plus years since diagnosis	0.846	−0.007
Plus BMI (kg/m^2^)	0.843	−0.003
Plus HbA_1c_ (mmol/mol)	0.838	−0.005

The respective ROC AUC scores illustrate the impact on performance when each variable is added to the model

Additional variables contributed only marginal improvements (ROC AUC gains of <0.02). For instance, including time since the last insulin dose >3.5 h increased the ROC AUC score by 0.011. The model reached its peak performance (ROC AUC of 0.873) with the inclusion of seven further variables. Beyond this point, adding more predictors either failed to enhance or slightly decreased performance, highlighting the limited utility of increased model complexity.

Based on these findings, three key variables – starting glucose, exercise duration and glucose trend arrows (classified as either falling or stable/rising) – were selected for building the simplified XGBoost model. This combination retained high predictive accuracy while facilitating the development of a practical, user-friendly visual decision support tool.

### Model performance: complex vs simplified model

The comprehensive XGBoost model incorporating all 406 variables (‘complex model’) demonstrated excellent performance in predicting exercise-associated hypoglycaemia, with a ROC AUC of 0.89 (95% CI 0.89, 0.90), sensitivity of 0.81 (95% CI 0.79, 0.82) and specificity of 0.80 (95% CI 0.78, 0.82) (Fig. 1a, blue line; Table 3).

**Table 3:** Performance of complex and simplified XGBoost models for predicting hypoglycaemia during exercise

Validation method	Dataset	Model	ROC AUC	Sensitivity	Specificity
*k*-fold cross-validation	T1DEXI	Complex XGBoost	0.89 (0.89, 0.90)	0.81 (0.79, 0.82)	0.80 (0.78, 0.82)
*k*-fold cross-validation	T1DEXI	Simplified XGBoost	0.87 (0.86, 0.88)	0.77 (0.77, 0.79)	0.78 (0.77, 0.79)
External validation	T1DEXIP	Simplified XGBoost	0.88 (0.87, 0.88)	0.78 (0.77, 0.79)	0.78 (0.77, 0.79)
External validation	EXT-101	Simplified XGBoost	0.84 (0.83, 0.84)	0.73 (0.72, 0.74)	0.72 (0.71, 0.73)
External validation	EXT-EDU	Simplified XGBoost	0.85 (0.85, 0.86)	0.76 (0.74, 0.78)	0.75 (0.74, 0.76)

Values represent the mean (95% CI) for ROC AUC, sensitivity and specificity. Confidence intervals were derived from 10-fold cross-validation

The results shown are for internal validation (10-fold cross-validation on the T1DEXI dataset) and external validation across three independent cohorts (T1DEXIP, EXT-EDU and EXT-101)

The simplified model, using only three easily accessible variables (starting glucose, exercise duration and glucose trend arrows) achieved a ROC AUC of 0.87 (95% CI 0.86, 0.88), with sensitivity of 0.77 (95% CI 0.77, 0.79) and specificity of 0.78 (95% CI 0.77, 0.79) (Fig. 1a, red line; Table 3). This represents only a modest reduction in predictive performance compared with the complex model.

The boxplot in Fig. 1b illustrates the distribution of predicted probabilities for hypoglycaemia across the two outcomes: ‘no hypoglycaemia’ and ‘hypoglycaemia’. The hypoglycaemia group shows a greater spread of predicted probabilities compared with the tightly clustered, low probabilities in the no hypoglycaemia group. Predicted probabilities are consistently higher for the hypoglycaemia group, with minimal overlap between IQRs, indicating strong model discrimination. The large number of outliers in the no hypoglycaemia group arises from the substantially larger number of non-hypoglycaemic bouts, leading to a wider spread of predicted probabilities.

The calibration curves in ESM Fig. 3 show how accurately the probability estimates of each model reflect the actual observed risk of hypoglycaemia in the data. The complex model (ESM Fig. 3, blue line) is generally well-calibrated, particularly at lower risk levels. At higher probability levels, it begins to show slight deviations, tending to overestimate the risk of hypoglycaemia. The simplified model (ESM Fig. 3, red line) demonstrates better calibration, aligning closely with the ideal calibration line across the full range of predicted probabilities. This suggests that the simplified model provides highly reliable probability estimates that more accurately reflect the actual risk of hypoglycaemia, and represent more interpretable and reliable risk estimates for clinical decision-making.

The SHAP dependence plots in ESM Figs 4–6 show the relationships between clinically relevant features and hypoglycaemia risk. These plots replicate patterns observed in previous studies, such as the effect of starting glucose, exercise duration, type and intensity of exercise, while also highlighting the nonlinear contributions of many variables, validating XGBoost as an appropriate modelling approach (ESM Results).

### Subgroup performance and calibration

**Fig. 2:**
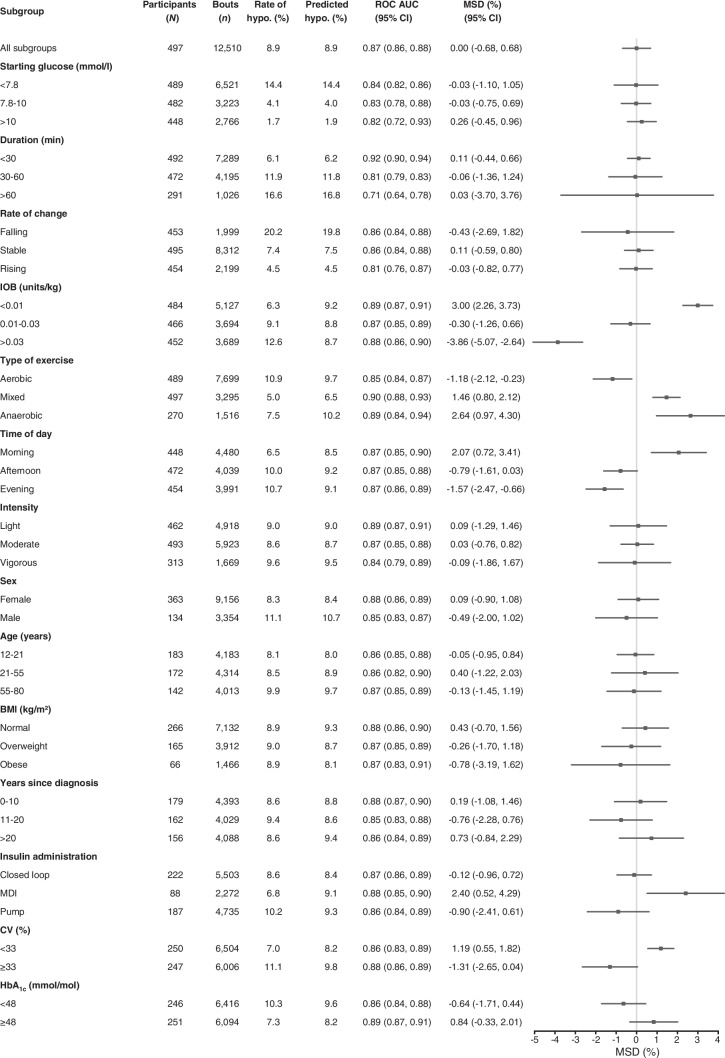
Subgroup analysis of the simplified XGBoost model. Each bar represents the MSD for the respective subgroup, with error bars indicating the 95% CIs. Rate of hypo. (%) denotes the percentage of bouts in which hypoglycaemia occurred. Predicted hypo. (%) indicates the mean predicted percentage of bouts with hypoglycaemia for each subgroup. MSD represents the mean signed difference between the observed and predicted hypoglycaemia rates, providing an indication of the direction and magnitude of average prediction bias. 'All subgroups' shows the overall scores for the model

Subgroup analysis of the simplified model (Fig. 2) showed that hypoglycaemia rates varied by factors including starting glucose, rate of change, exercise duration, IOB, exercise type and time of day. Despite this, the model remained well-calibrated, with predicted rates closely matching observed outcomes (mean signed deviation [MSD] 0.00%; 95% CI −0.68%, 0.68%).

The model was well-calibrated across most demographic and clinical variables, including age, sex and BMI. Minor discrepancies were observed: for IOB, the model slightly underpredicted risk at high IOB levels (MSD −3.86%) and overpredicted risk at low IOB levels (MSD +3.00%); for exercise type, underprediction occurred with aerobic activity (MSD −1.18%), and overprediction occurred with anaerobic (MSD +2.64%) and mixed exercise (MSD +1.46%); for time of day, risk was slightly underpredicted for exercise in the afternoon (MSD −0.79%) and evening (MSD −1.57%), and overpredicted for exercise in the morning (MSD +2.07%).Model accuracy was highly consistent across subgroups, but varied with exercise duration. While the model performed well across different exercise durations, the predictive performance was highest for bouts under 30 min (ROC AUC 0.92; 95% CI 0.90, 0.94) and decreased for sessions lasting 30–60 min (ROC AUC 0.81; 95% CI 0.79, 0.83) and over 60 min (ROC AUC 0.71; 95% CI 0.64, 0.78).

### Validation of the simplified model in independent cohorts

**Fig. 3:**
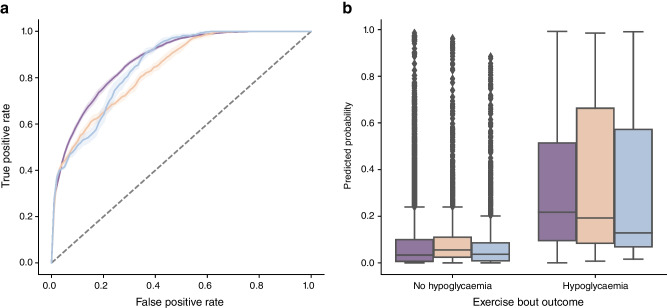
Validation of the simplified XGBoost model across independent cohorts. (**a**) ROC curves for the simplified model in three external validation cohorts: T1DEXIP (purple), EXT-101 (orange), and EXT-EDU (blue), with shaded areas indicating 95% CI. The dashed line indicates the mean decision threshold used for binary classification. (**b**) Boxplots showing the distribution of predicted hypoglycaemia probabilities for individual exercise bouts, stratified by observed outcome, across the three validation cohorts. The horizontal line represents the median, boxes indicate the IQR, whiskers extend to 1.5 × IQR, and diamond symbols indicate outliers

The simplified model was validated across three independent cohorts (T1DEXIP, EXT-101 and EXT-EDU), demonstrating strong and consistent performance, with ROC AUC values ranging from 0.83 to 0.88 (Fig. 3a and Table 3). The highest discrimination was seen in the T1DEXIP cohort (ROC AUC 0.88, 95% CI 0.87, 0.88). Both EXTOD cohorts also showed high performance (EXT-EDU: ROC AUC 0.85; 95% CI 0.85, 0.86; EXT-101: ROC AUC 0.84; 95% CI 0.84, 0.84).

The boxplot in Fig. 3b once again confirms clear separation in predicted probabilities between hypoglycaemia and non-hypoglycaemia events. The calibration curves in ESM Fig. 7 show accurate probability estimates across all cohorts, with only minor overestimation of risk in the EXT-101 cohort at higher probability levels.

### Translation of simple model to a user-friendly heatmap

The simplified model was translated into a practical, user-friendly heatmap designed to support real-time clinical and patient decision-making (ESM Methods). The tool displays predicted hypoglycaemia risk based on three variables: starting glucose, exercise duration (capped at 60 min due to reduced model performance beyond this) and glucose trend arrows (classified as either falling or stable/rising).

**Fig. 4:**
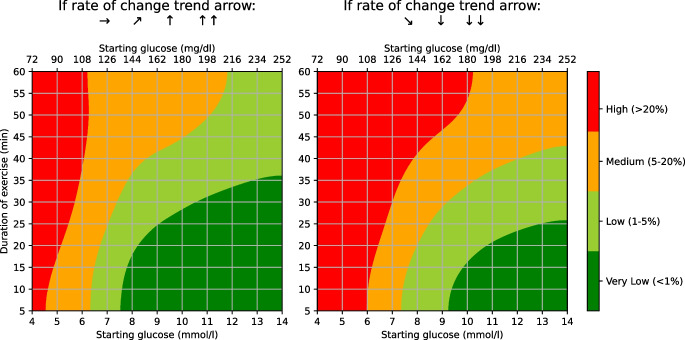
GlucoseGo heatmap showing the predicted risk of hypoglycaemia during exercise based on the simplified XGBoost model. The heatmap is divided into two panels according to glucose trend arrows: the left panel shows bouts with stable or rising glucose, and the right panel shows bouts with falling glucose. Colour-coded risk categories are represented as follows: dark green (<1% risk), light green (1–5% risk), amber (5–20% risk) and red (>20% risk)

Developed in collaboration with people with type 1 diabetes and healthcare professionals working with people with type 1 diabetes, the final heatmap uses a four-colour traffic-light system: very low risk (<1%; dark green), low risk (1–5%; light green), moderate risk (5–20%; amber) and high risk (>20%; red). This format was favoured for its clarity and ease of use. The final tool, named the GlucoseGo heatmap, is presented in Fig. 4.

The heatmap showed excellent calibration. Observed hypoglycaemia rates aligned well with predicted risk categories: 0.2% for very low risk (<1%; dark green), 2.2% for low risk (1–5%; light green), 11.5% for moderate risk (5–20%; amber) and 43.0% for high risk (>20%; red) (ESM Fig. 8). Calibration remained consistent across the variable that showed the biggest variation in subgroup analysis (IOB/kg, type of exercise, time of day and glycaemic variability 1 h prior) (ESM Fig. 9), supporting the robustness and clinical utility of the tool.

## Discussion

In this study, we developed and validated a simplified, user-friendly tool, GlucoseGo, for predicting the risk of exercise-associated hypoglycaemia in individuals over 12 years old with type 1 diabetes. Leveraging large, diverse datasets from four independent cohorts and over 16,000 recorded exercise bouts, we demonstrated that a model based on just three routinely available variables (starting glucose, exercise duration and glucose trend arrows), achieved predictive performance comparable to that of a complex model incorporating more than 400 variables. Through collaboration with individuals with type 1 diabetes, we translated this simplified model into the GlucoseGo heatmap: an intuitive, visual tool based on a traffic-light system that communicates hypoglycaemia risk prior to exercise. The heatmap demonstrated strong calibration, with observed hypoglycaemia rates aligning closely with predicted risk categories, and consistent performance across clinical variables and population subgroups.

Consistent with previous studies [7–9], our findings confirm starting glucose as the strongest predictor of exercise-associated hypoglycaemia. Inclusion of exercise duration and glucose trend arrows further enhanced the performance of the model while ensuring that it remained practical for real-world use. Unlike existing ML models [7–9], which often rely on complex or specialised data (e.g. the insulin on board level determined using a linear degradation algorithm, a low blood glucose index 24 h previously as obtained from CGM data, cyclic encoding of the hour of the day), our model balances usability with accuracy. Notably, only one prior study [9] has produced a similarly simple tool, although its applicability is limited due to a small sample size and controlled exercise conditions, which do not adequately capture the breadth of exercise activities undertaken in day-to-day life by people with type 1 diabetes.

SHAP analysis confirmed the model’s reliance on starting glucose, exercise duration and rate of change arrows as dominant predictors of exercise-associated hypoglycaemia, consistent with prior evidence [32–36]. In contrast, exercise type and intensity contributed minimally to the model’s predictions, which was an unexpected finding given their well-established physiological relevance. This discrepancy may reflect anticipatory behaviours by individuals, such as insulin dose adjustments or carbohydrate intake, based on the anticipated nature of the activity. It may also relate to the inherent difficulty of capturing these variables accurately in free-living settings, in which exercise bouts often include mixed modes and fluctuating intensities, and in which use of self-reported measures can introduce noise. These findings highlight the value of using data-driven approaches to reveal patterns that may differ from traditional clinical assumptions, and underscore the importance of real-world behavioural factors in determining hypoglycaemia risk.

The simplified model demonstrated strong calibration and generalisability across three independent cohorts and a wide range of clinical subgroups, including adolescents and individuals using MDI, insulin pumps or closed-loop systems. Model performance remained robust across most settings; however, predictive accuracy decreased for exercise bouts exceeding 60 min. Minor miscalibrations were observed: the model tended to slightly underestimate hypoglycaemia risk during aerobic activity, afternoon and evening exercise and when IOB was high, while overestimating risk for anaerobic activity, morning exercise and low IOB levels. These factors are important for clinicians, educators and individuals with type 1 diabetes to consider when interpreting the results. Nevertheless, the model’s overall calibration and consistency across diverse populations support its use in most routine exercise decisions. By relying on just three easily accessible variables – starting glucose, glucose trend (rate of change) and expected exercise duration – the tool offers a practical, accurate and interpretable means of assessing hypoglycaemia risk prior to exercise.

The reduction in predictive accuracy for bouts longer than 60 min probably reflects a general feature of forecasting models: predictive certainty decreases as the prediction window extends, as more uncontrolled variables can influence outcomes over time. This is analogous to weather forecasting: short-term predictions (e.g. for the next hour) are highly reliable, while those made several weeks in advance are inherently less accurate due to accumulating uncertainty. In our dataset, longer exercise sessions were also less frequent (*n*=1970), which may have contributed to greater variability and slightly less stable model performance.

The GlucoseGo heatmap holds significant clinical promise. Its intuitive traffic-light design, co-developed with individuals with type 1 diabetes, may support decision-making before exercise and help to reduce the fear of hypoglycaemia, which is a major barrier to physical activity in type 1 diabetes. Importantly, the heatmap aligns with clinical guidelines, using a threshold of 7 mmol/l as the recommended minimum glucose level for initiating exercise without additional carbohydrate intake [37]. This practical format may also alleviate some of the psychological burden of diabetes self-management (often referred to as ‘diabetes burnout’).

However, it is important to note that the heatmap's development used data from free-living exercise bouts, whereby participants may have already adjusted their insulin or device settings to account for activity. Therefore, while the GlucoseGo heatmap is a valuable tool, it is meant to complement, not replace, existing guidelines. After their usual exercise preparations, individuals can refer to the heatmap to assess whether further measures are necessary to avoid hypoglycaemia, enhancing the safety of their exercise and their experience when exercising.

Major strengths of this study are that our dataset included over 16,000 free-living exercise bouts of varying type (aerobic, anaerobic and mixed), participants with a wide age range (12–80 years), and individuals using a variety of insulin delivery methods (MDI, insulin pump and closed-loop). In addition, rather than limiting the dataset by imposing strict criteria, we tried to use as much data as possible to ensure that we trained the ML algorithms on the most diverse dataset, as this has been shown to produce more robust models [38–40].

Nevertheless, certain limitations should be acknowledged. The study population was predominantly white with relatively well-managed type 1 diabetes, with HbA_1c_ and time in range values close to guideline glycaemic targets (48 mmol/mol and 70%, respectively [31]), potentially limiting generalisability. The model has not yet been evaluated in children under 12 years of age. Data collection methods differed between cohorts (e.g. self-reported vs device-measured exercise intensity), and the retrospective design introduces the possibility of bias due to missing or inconsistent data. In addition, bolus insulin doses for MDI users were manually reported in the T1DEXI dataset, which may introduce inaccuracies in timing or omissions and therefore add noise to the IOB estimates. Furthermore, as the dataset reflects free-living behaviour, some participants may have adjusted their insulin or activity in ways that influenced the observed outcomes. It is also important to note that this model predicts hypoglycaemia risk during exercise only. Due to the physiological lag of CGM readings, individuals may still need to take action immediately after exercise to prevent hypoglycaemia as glucose levels continue to fall. Finally, as with all CGM-based studies, some symptomatic hypoglycaemia may not have been detected by the sensor, which represents an inherent limitation of the data.

Future research should prioritise validation in more diverse populations and in children under the age of 12 years, as there could be significant potential benefits for caregivers and educators in physical education settings. Prospective clinical trials are needed to determine the effectiveness of GlucoseGo in reducing hypoglycaemia and improving exercise engagement. Additional patient and healthcare professional involvement could further refine the tool’s usability and adoption in clinical practice.

In summary, GlucoseGo represents an important advance in the prediction of exercise-associated hypoglycaemia for people with type 1 diabetes. By distilling ML-derived predictions into a simple, user-friendly format, it supports informed decision-making and may promote physical activity and reduce hypoglycaemia burden. With further validation, this tool could help integrate personalised, data-driven support into routine diabetes self-management.

## Supplementary Information

Below is the link to the electronic supplementary material.

ESM (PDF 989 KB)

## Data Availability

The T1DEXI and T1DEXIP datasets are available through application to Vivli Inc. (https://vivli.org/). The EXTOD-101 and EXTOD-Education datasets are available from the study investigators upon reasonable request.

## Abbreviations

CGM: Continuous glucose monitor(ing)
EXTOD: EXercise in Type One Diabetes
EXT-101: EXTOD-101 study
EXT-EDU: EXTOD-Education study
IOB: Insulin units on board
JCHR: Jaeb Center for Health Research
MDI: Multiple daily injections
ML: Machine learning
MSD: Mean signed deviation
ROC: Receiver operating characteristics
SHAP: SHapley Additive exPlanations
T1DEXI: Type 1 Diabetes EXercise Initiative
T1DEXIP: Type 1 Diabetes EXercise Initiative Pediatric
XGBoost: eXtreme Gradient Boosting Algorithm

## Code availability

All code for this project is openly available in a GitHub repository (https://github.com/cafoala/glucoseGo).
