# Supplementary material for: GlucoseGo: a simple tool to predict hypoglycaemia during exercise in type 1 diabetes
Source: Diabetologia. 2026 Feb 25;69(6):1468–81. doi: 10.1007/s00125-026-06692-8 (PMC13109191; doi:10.1007/s00125-026-06692-8)
Supplement: Supplementary file 1 — ESM (PDF 989 KB) [file 125_2026_6692_MOESM1_ESM.pdf]

Electronic Supplementary Material (ESM)

ESM Methods

k-fold cross validation

The *k*-fold cross validation partitions data into 10 distinct subsets. For each iteration, a model is trained on 9 of these subsets and is validated on the remaining one, cycling through all subsets for comprehensive model validation [1, 2]. This results in 10 distinct models, with each data point appearing in exactly one test set.

Heatmap construction

To construct the heatmap, we used the simplified model (10 *k*-folds) to produce a mean risk score for each grid point in the range of 4-14 mmol/l (72-252 mg/dl) and durations from 5-60 min. These predictions were smoothed using a Gaussian filter [3] followed by bicubic interpolation [4] to reduce noise and smooth visualisation.

ESM Results

SHAP Analysis

The complex XGBoost relied largely on clinically relevant, explainable variables, with only three *tsfresh* variables identified within the top 12 most important variables in the SHAP summary plot (ESM Fig. 7). The eight most important, clinically relevant variables predicting risk of hypoglycaemia can be seen in the SHAP dependence plots in ESM Fig. 8. Starting glucose (ESM Fig. 8A) and exercise duration (ESM Fig. 8B) have the greatest predictive power and show that lower starting glucose and longer durations will increase the model's predicted risk of hypoglycaemia. Higher predicted risk of hypoglycaemia also appears to be associated with higher insulin on board (ESM Fig. 8C), aerobic exercise (ESM Fig. 8D), negative glucose rate of change (ESM Fig. 8H), and higher intensity exercise (ESM Fig. 8F). Lower risk appears to be associated with exercising in the morning (ESM Fig. 8E), and a bit less clearly, higher HbA_{1c} (ESM Fig. 8G), more years since diagnosis (ESM Fig. 8I), and higher BMI (ESM Fig. 8J).

However, while these patterns do appear within the data, the model does not establish simple linear relationships. Many variables exhibit non-linear effects, where their contribution to the model's prediction varies across different values. For example, variables such as HbA_{1c}, BMI, and years since diagnosis show highly non-linear patterns, making it difficult to extract a straightforward trend. Starting glucose, exercise duration, and IOB also display non-linear relationships, where their impact on predicted risk changes unevenly. This nonlinearity highlights the value of using a non-linear model, as it enables the capture of complex, nuanced patterns in the data that would be missed with a linear approach.

The difference in scale between the variables and thus the relative importance of these factors in prediction is very different: starting glucose and exercise duration have SHAP

values around five times greater than the fourth and fifth most important predictors (time of day and type of exercise).

ESM Tables

A)

Feature name	Description	Data type	Unique values	Missing values	Categories
intensity	The self-reported intensity of the exercise bout. 0=light, 1=moderate and 2=vigorous intensity.	Ordinal	4	0	0, 1, 2
day_of_week	The day of the week the exercise occurred. 0= Monday, 1=Tuesday, etc.	Categorical	7	0	0, 1, 2, 3, 4, 5, 6, 7
time_of_day	Time of day bout occurred. Morning 05:00-12:00, Afternoon 12:00-17:00, Evening 17:00-05:00	Categorical	3	0	'morning', 'afternoon', 'evening' ,
form_of_exercise	The predominant type of the exercise	Categorical	3	1	'aer', 'mix', 'ana'
sex	Sex	Categorical	2	0	'male' , 'female'
insulin_modality	Type of insulin administration	Categorical	3	11	'mdi' , 'pump' , 'closed_loop'
season	The season in which the exercise occurred	Categorical	4	0	0, 1, 2, 3
ID	Identifies participants using the study name and a unique number	Categorical	834	0	'dexip_100' … 'dexi_988'
stratify	Variable created to stratify the data based on ID and target variable	Categorical	1356	0	'dexip_100_False' ... 'dexi_988_True'
study	Which study the participant is in	Categorical	4	0	'dexi' 'dexip', 'ext_101', 'ext_edu',
bout_id	The ID for individual bouts, ID plus timestamp of bout start	Categorical	16577	0	'dexi_100_202106101306 00' ... 'dexi_988_202133120833 06'
y	Target variable – CGM dropped below 3.9mmol/l or 70mg/dl during exercise	Boolean	2	0	False, True

B)

Feature name	Description	Units	Missing values (N)	Min. value	Q1	Q2	Q3	Max. value

duration	Duration of exercise bout	min	0	10.0	22.0	30.0	48.0	120.0
start_glc	The last glucose reading before exercising	mmol/l	0	2.1	6.2	7.8	10.1	22.6
start_roc	The rate of change before the last glucose reading and the reading >20mins before that reading	mmol/l /hour	92	-23.1	-1.8	0.00	1.7	21.6
iob_kg	The insulin on board (IOB) per kg. Calculated using a linear degradation algorithm with duration of action set to 4 hours.	Units/ kg	N/A	0	0.00084	0.018	0.042	0.64
age	Participant's age	years	27	12.00	21.00	30.00	46.00	80.16
hba1c	HbA_{1c}	mmol/ mol	136	29.0	43.2	48.6	55.2	140.4
bmi	BMI	kg/m^2	39	13.7	21.6	23.7	26.3	48.5
years_since_diagnosis	Years since diagnosis	years	206	0.48	10.0	14.0	23.0	66.0

C)

	Description	Units	Missing values (N)	Min. value	Q1	Q2	Q3	Max. value
Data sufficiency	The percentage of data present (total expected CGM readings minus missing readings)	%	0	7.70	92.30	92.30	92.30	100.00
Average glucose	The mean glucose of all the readings	(mmol/l)	0	2.17	6.16	7.75	9.89	25.65
eA1c	Estimated A1C	%	0	2.99	5.51	6.50	7.85	17.76
SD	Standard deviation	(mmol/l)	11	0.00	0.34	0.59	1.00	5.23
CV	Coefficient of variation	%	11	0.00	4.27	7.43	12.82	72.26
AUC	Area under the curve	mmolh/L	15	0.31	2.76	3.76	5.52	20.42
LBGI	Low blood glucose index		0	0.00	0.00	0.00	0.31	38.34
HBGI	High blood glucose index		0	0.00	0.12	2.03	7.68	70.83
MAGE	Mean amplitude of glycemic excursions	mmol/l	28	0.05	0.83	1.50	2.61	14.05
TIR normal	Percentage time in range 3.9-10mmol/l	%	0	0.00	50.00	100.00	100.00	100.00

TIR normal 1	Percentage time in range 3.9-7.8mmol/l	%	0	0.00	0.00	50.00	100.00	100.00
TIR normal 2	Percentage time in range 7.8-10mmol/l	%	0	0.00	0.00	0.00	41.67	100.00
TIR level 1 hypoglycaemia	Percentage time in range 3.9-3.0mmol/l	%	0	0.00	0.00	0.00	0.00	100.00
TIR level 2 hypoglycaemia	Percentage time in range <3.0mmol/l	%	0	0.00	0.00	0.00	0.00	100.00
TIR level 1 hyperglycemia	Percentage time in range 10.0-13.9mmol/l	%	0	0.00	0.00	0.00	25.00	100.00
TIR level 2 hyperglycemia	Percentage time in range >13.9mmol/l	%	0	0.00	0.00	0.00	0.00	100.00
Total number hypoglycemic events	The number of times glucose drops below 3.9mmol/l for 15 mins or more	*N*	0	0.00	0.00	0.00	0.00	1.00
Number LV1 hypoglycemic events	The number of times glucose drops below 3.9mmol/l for 15 mins or more but not below 3.0mmol/l	*N*	0	0.00	0.00	0.00	0.00	1.00
Number LV2 hypoglycemic events	The number of times glucose drops below 3.0mmol/l for 15 mins or more	*N*	0	0.00	0.00	0.00	0.00	1.00
Number prolonged hypoglycemic events	The number of times glucose drops below 3.0mmol/l for 120 mins or more	*N*	0	0.00	0.00	0.00	0.00	0.00
Avg. length of hypoglycemic events	The mean length of all hypoglycemic episodes (anything below 3.9mmol/l)	min	0	0.00	0.00	0.00	0.00	55.08
Total time spent in hypoglycemic events	The combined length of all hypoglycemic episodes (anything below 3.9mmol/l)	min	0	0.00	0.00	0.00	0.00	55.08
Total number hyperglycemic events	The number of times glucose rises above 10.0mmol/l for 15 mins or more	min	0	0.00	0.00	0.00	1.00	2.00
Number LV1 hyperglycemic events	The number of times glucose rises above 10.0mmol/l for 15 mins or more but not above 13.9mmol/l	*N*	0	0.00	0.00	0.00	0.00	2.00
Number LV2 hyperglycemic events	The number of times glucose rises above 13.9mmol/l for 15 mins or more	*N*	0	0.00	0.00	0.00	0.00	1.00
Number prolonged hyperglycemic events	The number of times glucose rises above 13.9mmol/l for 120 mins or more	*N*	0	0.00	0.00	0.00	0.00	0.00

Avg. length of hyperglycemic events	The mean length of all hypoglycemic episodes (anything above 10.0mmol/l)	min	0	0.00	0.00	0.00	24.98	59.93
Total time spent in hyperglycemic events	The combined length of all hypoglycemic episodes (anything above 10mmol/l)	min	0	0.00	0.00	0.00	24.98	59.93

D: Tsfresh Features Extracted from 1-Hour Pre-Exercise CGM Data

- Variance larger than standard deviation
- Has duplicate maximum
- Has duplicate minimum
- Has duplicate values
- Sum of values
- Absolute energy
- Mean absolute change
- Mean change
- Mean second derivative (central)
- Median
- Mean
- Length (number of readings)
- Standard deviation
- Coefficient of variation
- Variance
- Skewness
- Kurtosis
- Root mean square
- Absolute sum of changes
- Longest strike below mean
- Longest strike above mean
- Count above mean
- Count below mean
- Last location of maximum
- First location of maximum
- Last location of minimum
- First location of minimum
- Percentage of reoccurring values to all values
- Percentage of reoccurring datapoints to all datapoints
- Sum of reoccurring values
- Sum of reoccurring datapoints
- Ratio of value number to time series length
- Maximum
- Absolute maximum
- Minimum
- Benford correlation
- Time reversal asymmetry statistic (lags 1, 2, 3)

- C3 statistic (lags 1, 2, 3)
- Complexity-invariant distance (with and without normalization)
- Symmetry looking (various r values)
- Large standard deviation (various r values)
- Quantiles (10%, 20%, 30%, 40%, 60%, 70%, 80%, 90%)
- Autocorrelation (lags 0–9)
- Aggregated autocorrelation (mean, median, variance, max lag 40)
- Partial autocorrelation (lags 0–5)
- Number of CWT peaks (n=1, 5)
- Number of peaks (n=1, 3, 5, 10, 50)
- Binned entropy (10 bins)
- Index mass quantiles (various quantiles)
- Continuous wavelet transform (CWT) coefficients (various coefficients and widths)
- Welch's density coefficients (coeffs 2, 5)
- AR coefficients (coeff 10, k=10)
- Change quantiles (various quantiles and aggregation functions)
- Fast Fourier transform (FFT) coefficients (real, imaginary, absolute, angle, various coeffs)
- Aggregated FFT features (centroid, variance)
- Value count for specific values (0, 1, -1)
- Range count for specific max/min
- Approximate entropy (various m and r)
- Linear trend (p-value, r-value, intercept, slope, stderr)
- Aggregated linear trend (r-value, intercept, slope, stderr; chunk lengths 5/10; max, min, mean, variance)
- Augmented Dickey-Fuller test (test statistic, p-value, used lag)
- Number crossing mean (for various m)
- Energy ratio by chunks (num segments 10, segment focus 0–9)
- Ratio beyond r sigma (various r values)
- Count above a threshold (t=0)
- Count below a threshold (t=0)
- Lempel-Ziv complexity (various bin counts)
- Fourier entropy (various bin counts)
- Permutation entropy (dimension 3–7, tau=1)
- Mean of the n absolute maxima (n=7)

ESM Table 1. Data Dictionary Describing All Variables Used in Analysis.

A: Demographic, lab, and exercise data for categorical variables B: Demographic, lab, and exercise data for numerical variables. C: Metrics of glycemic control from CGM data one hour prior to exercise. D: List of time-series features extracted using tsfresh from the CGM trace in the hour preceding exercise.

k-fold number	1	2	3	4	5	6	7	8	9	10
objective	binary:logistic	binary:logistic	binary:logistic	binary:logistic	binary:logistic	binary:logistic	binary:logistic	binary:logistic	binary:logistic	binary:logistic
base_score	0.5	0.5	0.5	0.5	0.5	0.5	0.5	0.5	0.5	0.5
booster	gbtree	gbtree	gbtree	gbtree	gbtree	gbtree	gbtree	gbtree	gbtree	gbtree
colsample_bylevel	1	1	1	1	1	1	1	1	1	1
colsample_bynode	1	1	1	1	1	1	1	1	1	1
colsample_bytree	0.760	0.519	0.802	0.832	0.774	0.870	0.803	0.818	0.973	0.822
gamma	2	4	4	0	4	4	0	4	2	1
learning_rate	0.164	0.081	0.137	0.101	0.169	0.116	0.086	0.085	0.137	0.092
max_delta_step	0	0	0	0	0	0	0	0	0	0
max_depth	8	9	9	8	5	4	8	10	12	7
min_child_weight	8	7	9	7	8	1	8	5	7	8
n_estimators	236	588	976	312	7	282	233	219	389	908
n_jobs	12	12	12	12	12	12	12	12	12	12
num_parallel_tree	1	1	1	1	1	1	1	1	1	1
predictor	auto	auto	auto	auto	auto	auto	auto	auto	auto	auto
random_state	42	42	42	42	42	42	42	42	42	42
reg_alpha	4	3	1	2	1	2	1	1	3	1
reg_lambda	0	0	1	4	2	3	0	4	2	1
scale_pos_weight	1	1	1	1	1	1	1	1	1	1
subsample	0.866	0.871	0.734	0.692	0.993	0.997	0.933	0.991	0.801	0.860
tree_method	exact	exact	exact	exact	exact	exact	exact	exact	exact	exact
validate_parameters	1	1	1	1	1	1	1	1	1	1
eta	0.234	0.157	0.244	0.131	0.211	0.155	0.163	0.219	0.154	0.161
seed	42	42	42	42	42	42	42	42	42	42

ESM Table 2. Tuned hyperparameter for the 10 k-fold models for the complex XGBoost.

k-fold number	1	2	3	4	5	6	7	8	9	10
objective	binary:logistic	binary:logistic	binary:logistic	binary:logistic	binary:logistic	binary:logistic	binary:logistic	binary:logistic	binary:logistic	binary:logistic
base_score	0.5	0.5	0.5	0.5	0.5	0.5	0.5	0.5	0.5	0.5
booster	gbtree	gbtree	gbtree	gbtree	gbtree	gbtree	gbtree	gbtree	gbtree	gbtree
colsample_bylevel	1	1	1	1	1	1	1	1	1	1
colsample_bynode	1	1	1	1	1	1	1	1	1	1
colsample_bytree	0.99	0.77	0.94	0.89	0.88	0.69	0.83	0.82	0.72	0.89
enable_categorical	FALSE	FALSE	FALSE	FALSE	FALSE	FALSE	FALSE	FALSE	FALSE	FALSE
gamma	4	4	4	3	0	4	5	5	4	1
gpu_id	-1	-1	-1	-1	-1	-1	-1	-1	-1	-1
learning_rate	0.48	0.19	0.36	0.11	0.27	0.24	0.24	0.31	0.15	0.22
max_delta_step	0	0	0	0	0	0	0	0	0	0
max_depth	8	5	8	3	5	3	9	4	11	3
min_child_weight	5	7	5	1	10	2	8	10	7	1
n_estimators	325	867	586	637	489	135	49	322	584	507
n_jobs	12	12	12	12	12	12	12	12	12	12
num_parallel_tree	1	1	1	1	1	1	1	1	1	1
predictor	auto	auto	auto	auto	auto	auto	auto	auto	auto	auto
random_state	42	42	42	42	42	42	42	42	42	42
reg_alpha	5	1	0	4	2	1	0	1	1	2
reg_lambda	3	3	5	3	5	5	1	4	3	1
scale_pos_weight	1	1	1	1	1	1	1	1	1	1
subsample	0.832	0.905	0.920	0.959	0.582	0.699	0.696	0.893	0.602	0.779
tree_method	exact	exact	exact	exact	exact	exact	exact	exact	exact	exact
validate_parameters	1	1	1	1	1	1	1	1	1	1
eta	0.142	0.262	0.117	0.191	0.263	0.155	0.254	0.117	0.114	0.128
seed	42	42	42	42	42	42	42	42	42	42

ESM Table 3. Tuned hyperparameter for the 10 k-fold models for the simplified XGBoost.

ESM Figures

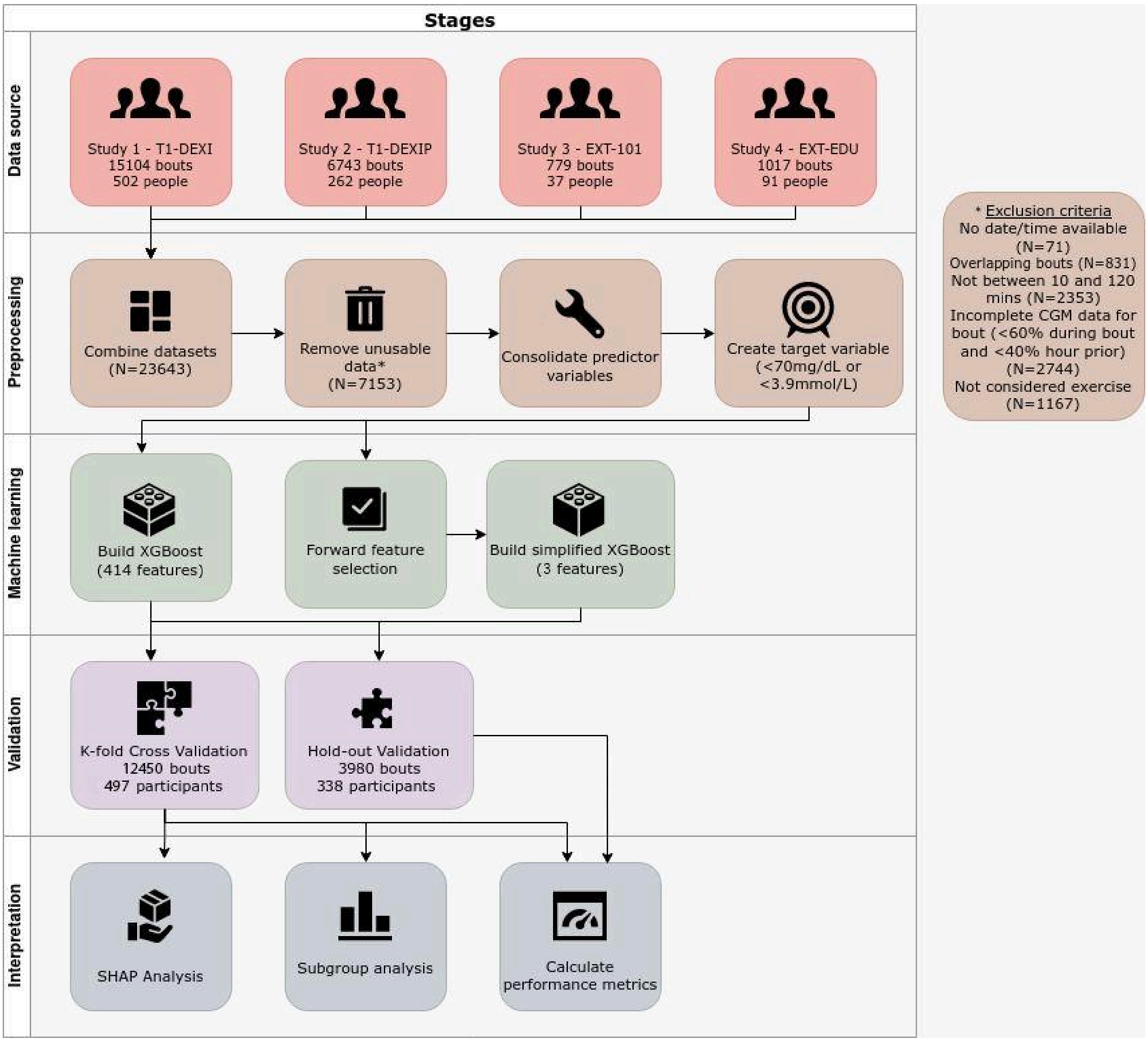

ESM Fig. 1. Graphical representation of modelling method, including data exclusions.

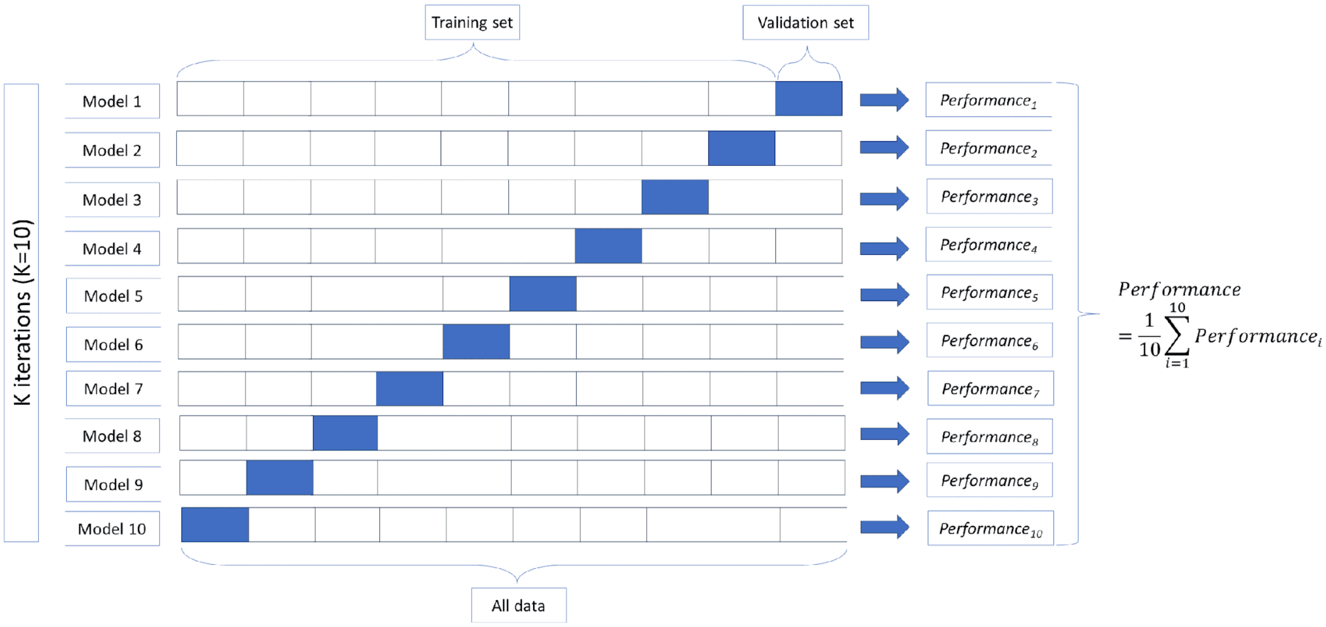

ESM Fig. 2. *k*-fold cross validation. Visual representation of *k*-fold cross validation (k=10).

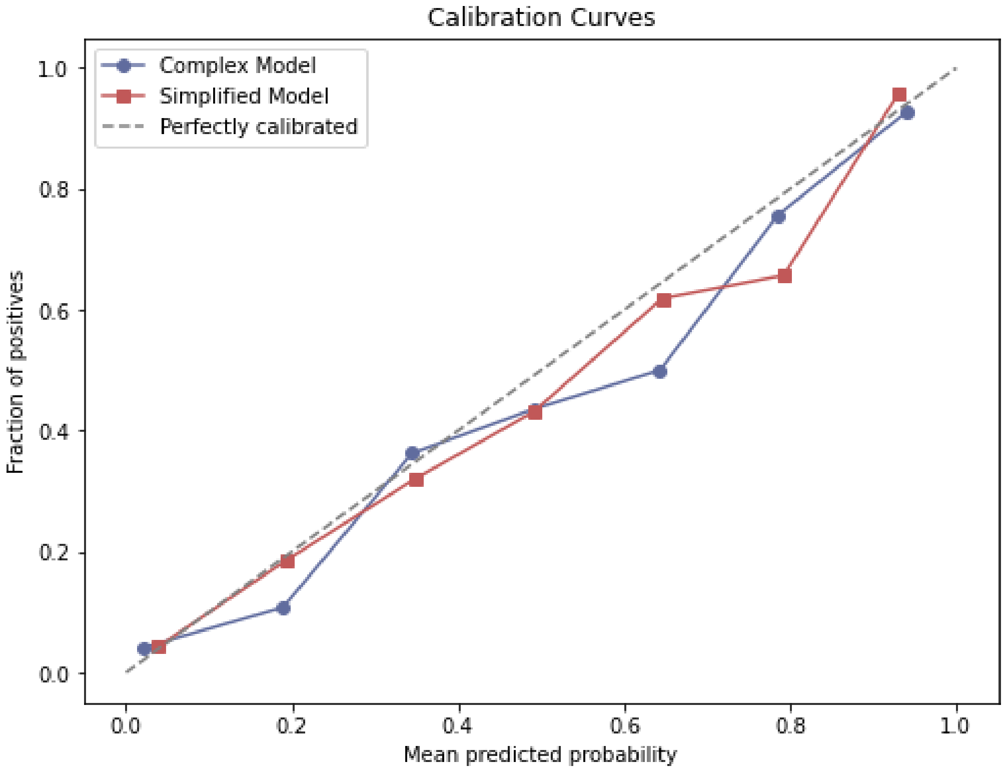

ESM Fig. 3. Calibration curves for the complex and simplified models in the T1DEXI dataset. *The plot shows the calibration curves for the complex (blue line) and simplified (red line) XGBoost models using the T1DEXI dataset. The x-axis represents the predicted probability of hypoglycaemia during exercise, while the y-axis shows the observed proportion of exercise bouts that actually resulted in hypoglycaemia. The dashed diagonal line indicates perfect calibration, where predicted probabilities exactly match observed outcomes. Both models are generally well-calibrated, but the simplified model closely follows the ideal line across the full range of probabilities.*

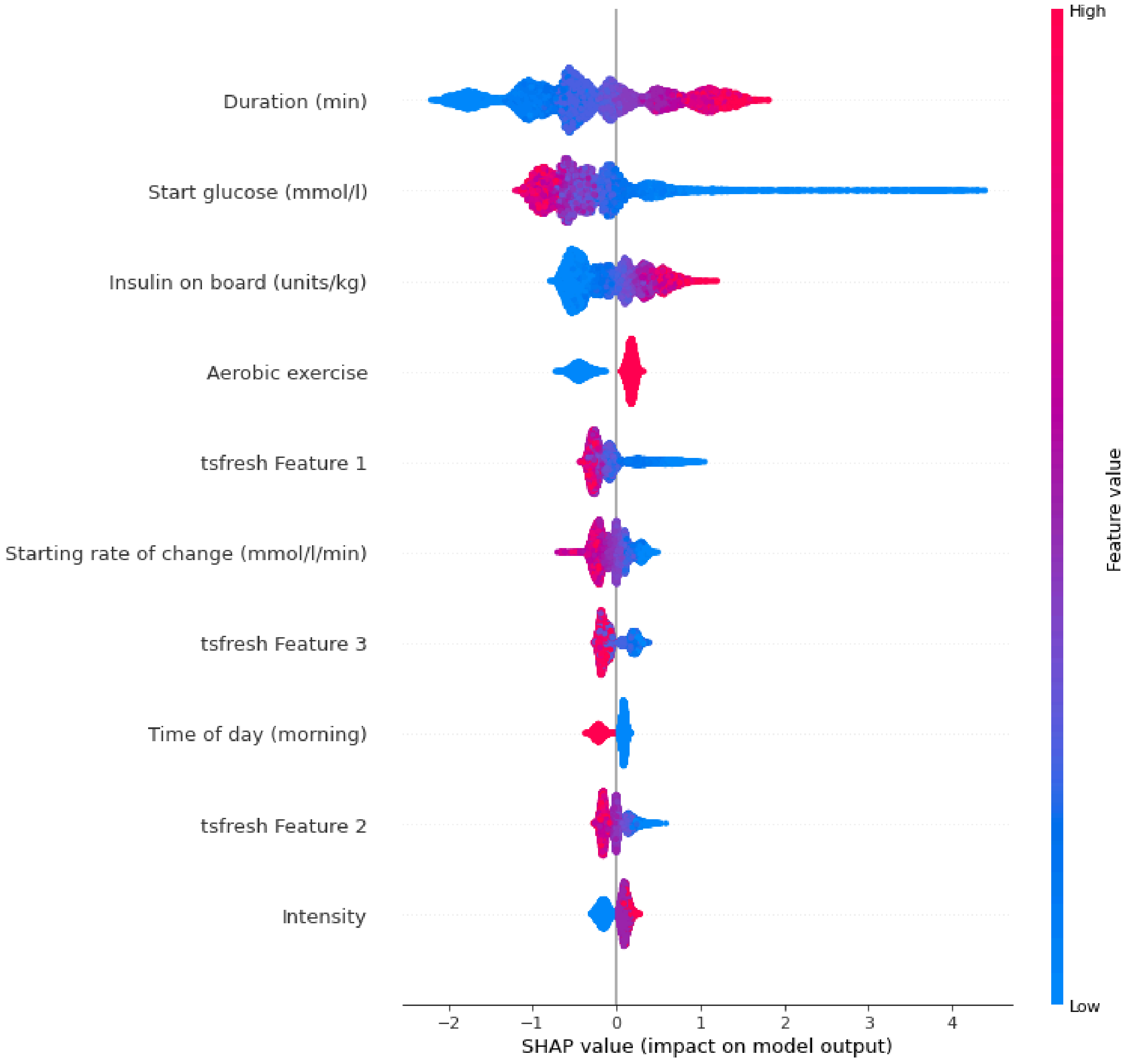

ESM Fig. 4. SHAP analysis for complex XGBoost model (all features). *SHAP values illustrate the contribution of each feature to the final prediction, shown as a shift in log-odds. Negative values indicate a decreased risk of hypoglycaemia, and positive values an increased risk. Feature values are colour-coded from blue (low) to red (high). Features are ordered (top to bottom) by their overall average contribution to model predictions.*

tsfresh features extracted from the one hour of CGM data prior to exercise. tsfresh Feature 1 represents the Continuous Wavelet Transform (CWT) coefficient. It computes the wavelet coefficients for various widths, specifically accessing the 11th coefficient. tsfresh Feature 2 computes the average value of the central second derivative, which is an approximation of the curvature or concavity of the time series. tsfresh Feature 3 extracts the angle (phase) of a coefficient from the Fast Fourier Transform (FFT) of the time series.

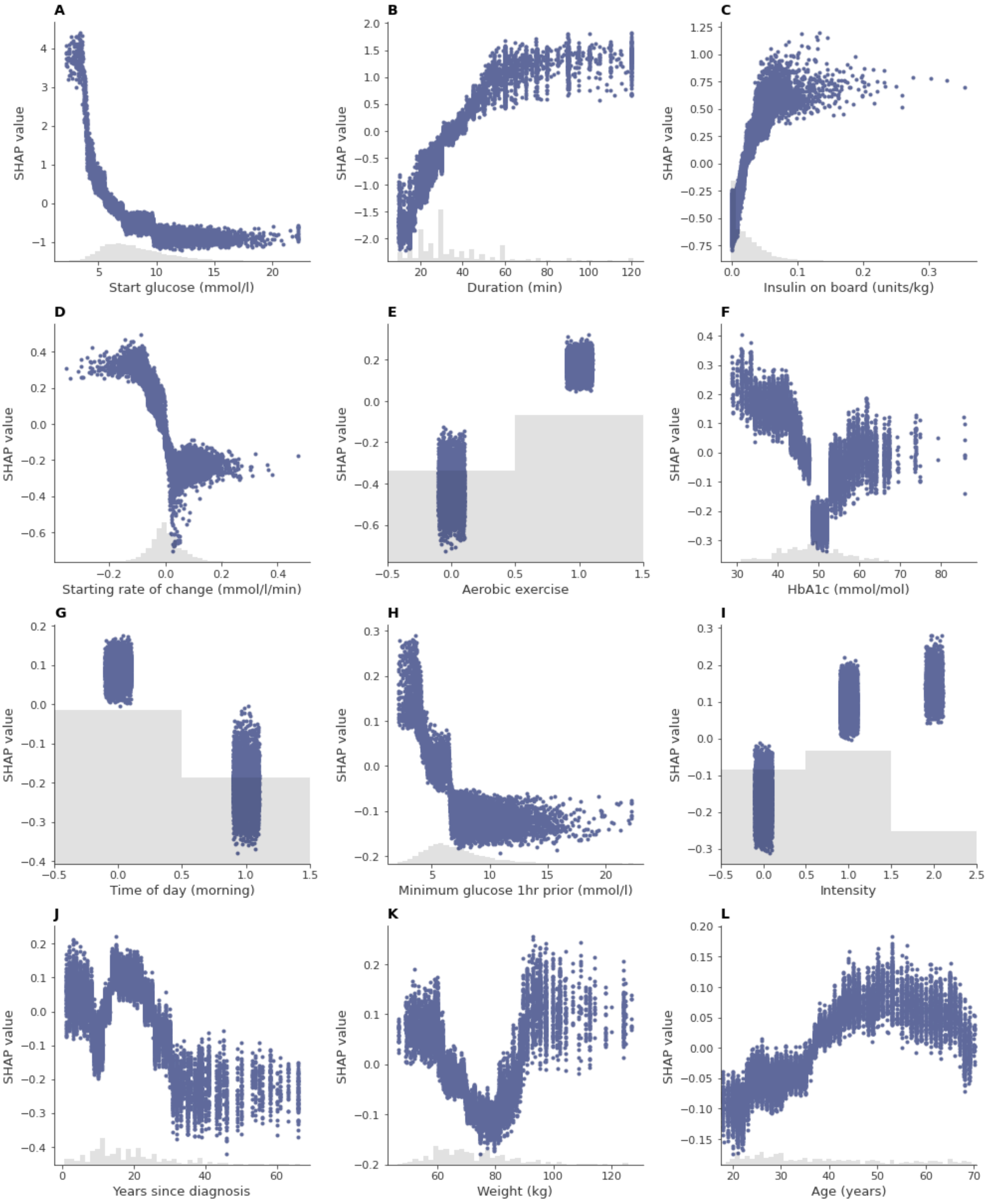

ESM Fig. 5. SHAP dependence plots for clinically relevant features from the complex XGBoost model. *Blue points indicate the SHAP value (on the y-axis) for any given variable value (x-axis). SHAP values illustrate the contribution of each variable to the final prediction, shown as a*

shift in log-odds. Negative values indicate a decreased risk of hypoglycaemia, and positive values an increased risk. The grey shaded area shows a histogram of variable values. The plot highlights the six most clinically relevant variables with the greatest overall average contribution to model predictions.

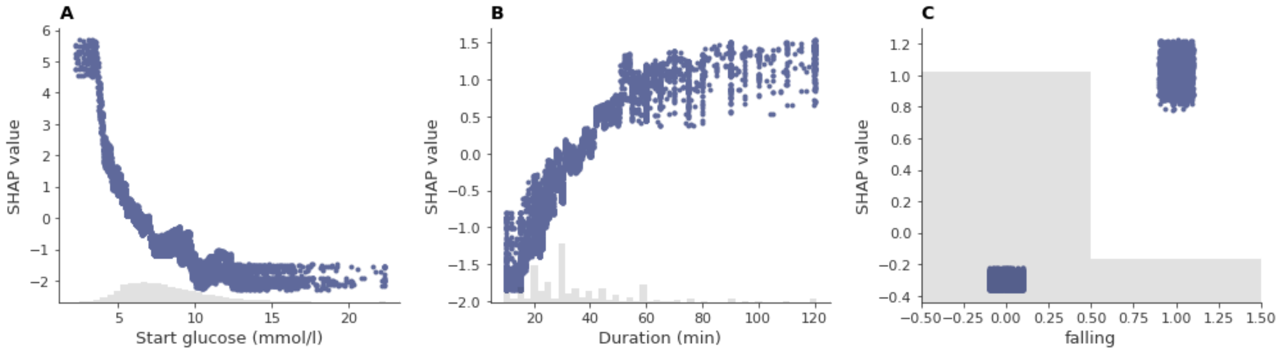

ESM Fig. 6. SHAP dependence plots for clinically relevant features from the simplified XGBoost model. *Blue points indicate the SHAP value (on the y-axis) for any given variable value (x-axis). SHAP values illustrate the contribution of each variable to the final prediction, shown as a shift in log-odds. Negative values indicate a decreased risk of hypoglycaemia, and positive values an increased risk. The grey shaded area shows a histogram of variable values. The plot highlights the three variables selected in feature selection. "Falling" is a binary variable that shows whether or not the rate of change was below 0.05 mmol/l / min (falling=1) or stable/rising (falling=0).*

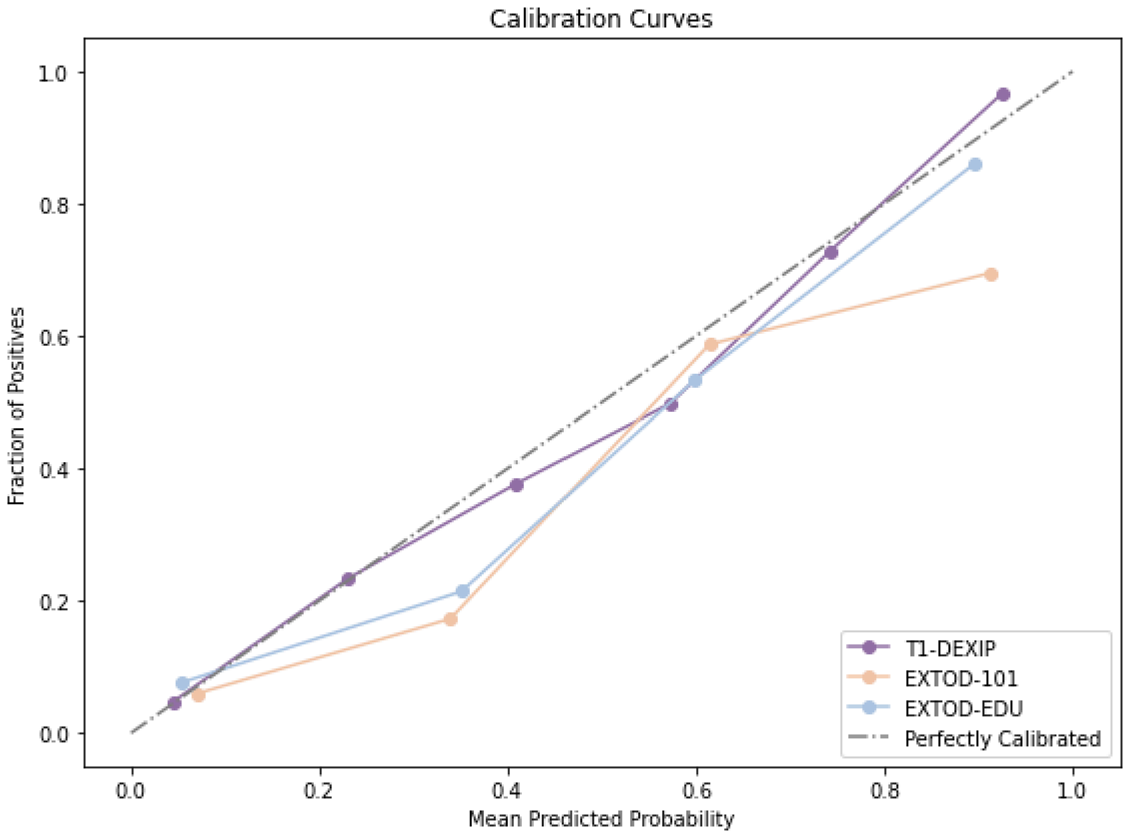

ESM Fig. 7. Calibration curves for the simplified model in external validation cohorts. *The plot displays calibration curves for the simplified model applied to three independent validation cohorts: T1-DEXIP (purple line), EXTOD-101 (beige line), and EXTOD-EDU (blue line). The x-axis indicates the predicted probability of hypoglycaemia, and the y-axis represents the observed proportion of hypoglycaemia events in each probability bin. The dashed diagonal line corresponds to perfect calibration. The model remains well-calibrated across all cohorts, with a slight tendency to overestimate risk at higher probabilities in the EXTOD-101 cohort.*

A

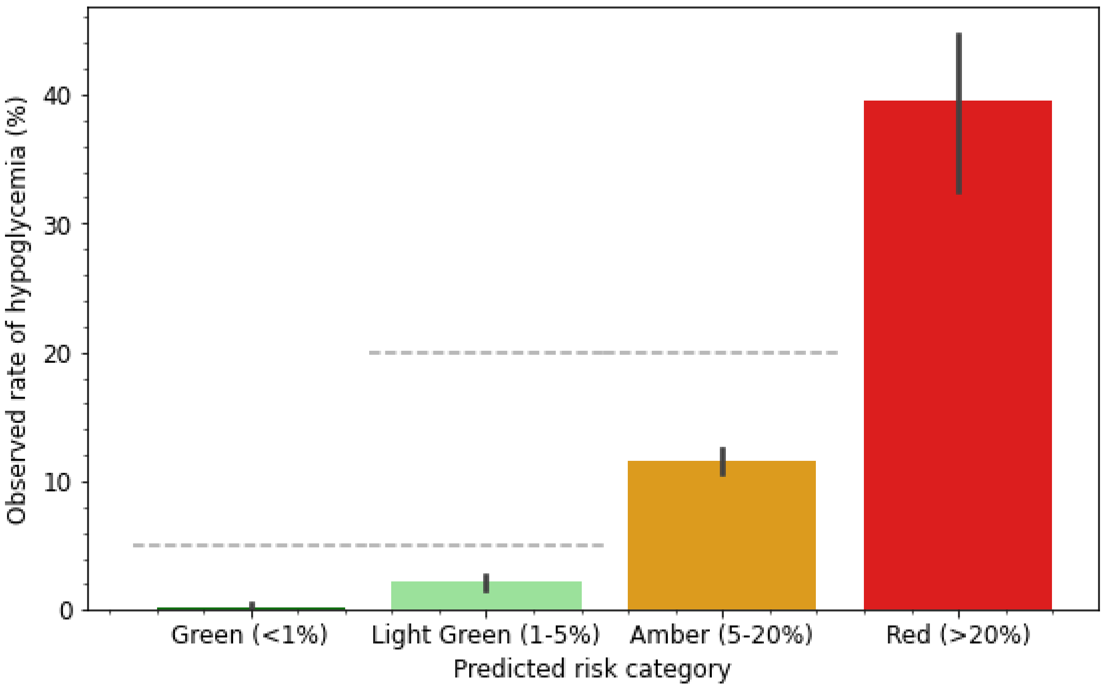

B

Predicted risk category	Observed rate of hypoglycemia (%)
Green (<1%)	0.2 (-0.0, 0.4)
Light Green (1-5%)	2.2 (1.6, 2.7)
Amber (5-20%)	11.5 (10.6, 12.4)
Red (>20%)	39.5 (32.8, 46.3)

ESM Fig. 8. Calibration of heatmap results. *A: The bar chart shows the actual frequency of hypoglycaemia in the three heat-map risk zones of <1% (green), 1-5% (light green), 5-20%, (amber), and >20% (red). The grey dotted lines show the 5% and 20% thresholds. The spots represent the result for each of the 10 k-folds. B: The predicted and observed rate of hypoglycaemia within the zones with the 95% confidence intervals.*

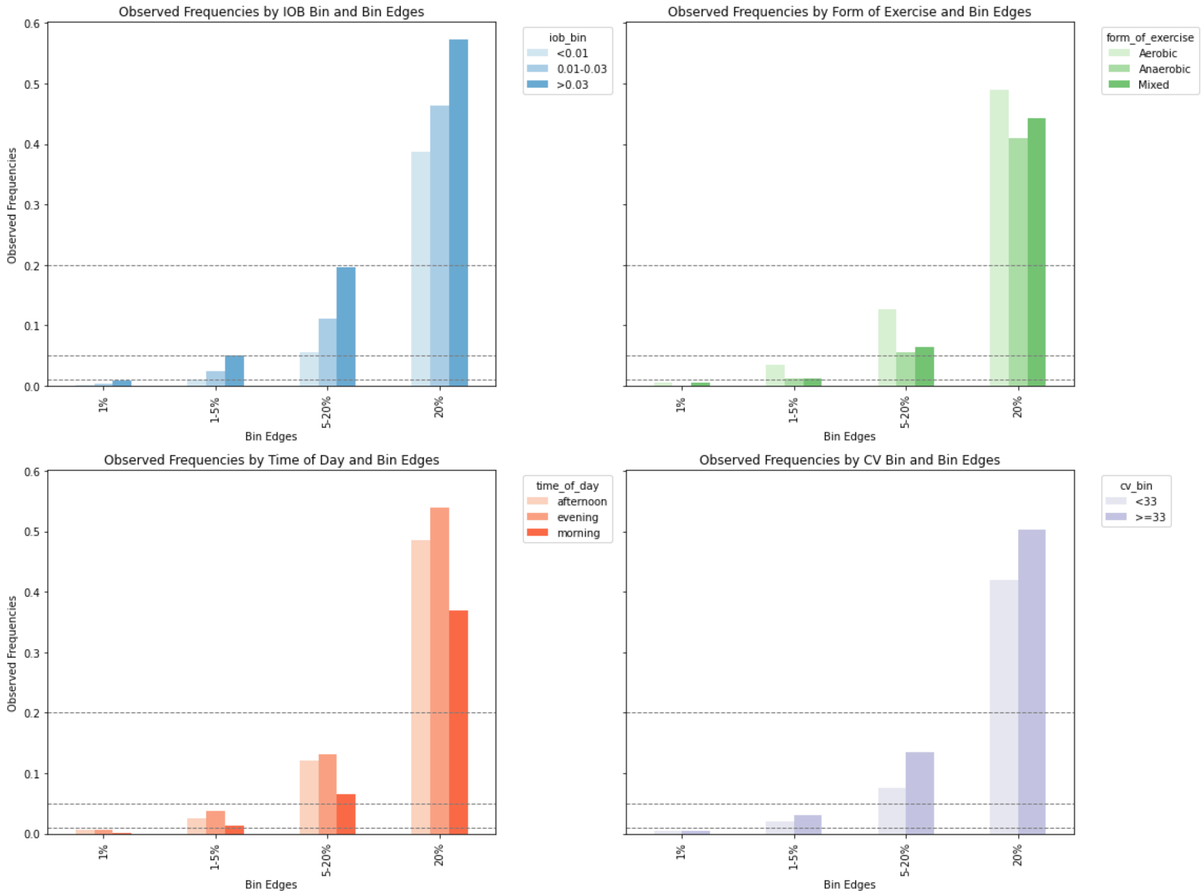

ESM Fig. 9. Calibration of the GlucoseGo heatmap across key subgroups. *Bar plots show the observed rate of hypoglycaemia during exercise for each heatmap risk category (<1%, 1-5%, 5–20%, >20%), stratified by (A) insulin on board (IOB), (B) predominant form of exercise, (C) time of day, and (D) coefficient of variation (CV). For each subgroup, the observed rates fall within the intended risk thresholds indicated by the heatmap categories, confirming that the model's predictions remain well-calibrated across variations in IOB, exercise type, time of day, and glycaemic variability. Dashed horizontal lines indicate the thresholds for the heatmap risk categories: <1%, 1-5%, 5–20%, and >20%.*

References

1. Wong TT, Yeh PY. Reliable accuracy estimates from k-fold cross-validation. *IEEE Trans Knowl Data Eng*. 2020;32(8):1586–1594. doi:10.1109/TKDE.2019.2912815

2. Yadav S, Shukla S. Analysis of k-fold cross-validation over hold-out validation on colossal datasets for quality classification. In: *Proc IEEE 6th Int Conf Adv Comput (IACC)*. 2016. p. 78–83. doi:10.1109/IACC.2016.25

3. Virtanen P, Gommers R, Oliphant TE, et al. SciPy 1.0: fundamental algorithms for scientific computing in Python. *Nat Methods*. 2020;17(3):261–272. doi:10.1038/s41592-019-0686-2

4. Hunter JD. Matplotlib: a 2D graphics environment. Comput Sci Eng. 2007;9(3):90–95. doi:10.1109/MCSE.2007.55
